# A Narrative Review on Abnormalities in the Hemostatic System in Diabetes Mellitus: Pathophysiology, Clinical Implications, and Therapeutics

**DOI:** 10.3390/life16040648

**Published:** 2026-04-12

**Authors:** Sana Rafaqat, Hafsa Hamid, Fakhra Bashir, Hijab Abaid, Aleksandra Klisic, Saira Rafaqat, Filiz Mercantepe

**Affiliations:** 1 Department of Biotechnology (Human Genetics), Lahore College for Women University, Lahore 44444, Pakistan; sana.rafaqat44@gmail.com; 2 Department of Biotechnology, Lahore College for Women University, Lahore 44444, Pakistan; 3 School of Biochemistry and Biotechnology, University of the Punjab, Lahore 54590, Pakistan; 4 Faculty of Medicine, University of Montenegro, 81000 Podgorica, Montenegro; 5 Center for Laboratory Diagnostics, Primary Health Care Center, 81000 Podgorica, Montenegro; 6 Department of Zoology, Lahore College for Women University, Lahore 44444, Pakistan; 7 Department of Endocrinology and Metabolism, Faculty of Medicine, Duzce University, Duzce 81620, Türkiye

**Keywords:** diabetes mellitus, type 1 diabetes, type 2 diabetes, gestational diabetes, coagulation biomarkers, endothelial dysfunction, fibrinolysis, cardiovascular complications

## Abstract

Diabetes mellitus (DM) is a complex metabolic disorder associated with a heightened risk of cardiovascular events, largely driven by a hypercoagulable and hypofibrinolytic state. The pathophysiological interplay between chronic hyperglycemia, oxidative stress, insulin resistance, and systemic inflammation fosters profound alterations in the coagulation cascade, endothelial function, and platelet activity. This narrative review synthesizes evidence from studies published between 2008 and 2026, focusing on coagulation and platelet-related biomarkers selected based on their biological relevance to thrombosis, endothelial dysfunction, and inflammation, as well as the availability of clinical and interventional data across different forms of DM. Although there are numerous biomarkers involved in the pathogenesis of various forms of diabetes, this narrative review critically examines key coagulation biomarkers—including D-dimer, fibrinogen, prothrombin, tissue thromboplastin or tissue factor, P-selectin, soluble urokinase plasminogen activator receptor, thrombomodulin, plasminogen activator inhibitor-1, von Willebrand factor, and β-thromboglobulin—across distinct diabetes subtypes, including type 1, type 2, gestational, and secondary forms linked to endocrinopathies and pancreatic diseases. The literature reveals substantial subtype-specific heterogeneity in hemostatic alterations. For instance, Type 1 DM is characterized by early endothelial dysfunction and platelet activation, while Type 2 DM presents with elevated coagulation factors, impaired fibrinolysis, and a proinflammatory milieu. Gestational DM exhibits pregnancy-specific changes in coagulation, yet distinguishing them from obesity-related effects remains challenging. Secondary diabetes forms, such as those associated with Cushing’s syndrome or pancreatitis, further underscore the diversity in thrombotic risk profiles. Among the coagulation and platelet activation biomarkers reviewed, fibrinogen, P-selectin, and plasminogen activator inhibitor-1 demonstrate the most consistent associations with glycemic control, vascular dysfunction, and therapeutic modulation, particularly in type 2 diabetes, suggesting greater potential for clinical translation. In contrast, evidence for markers such as D-dimer, tissue factor or tissue thromboplastin, and soluble urokinase plasminogen activator receptor remains heterogeneous and insufficient for routine clinical application. By synthesizing mechanistic insights and clinical data, this review highlights the urgent need for subtype-tailored coagulation assessment in diabetes management. A better understanding of the dynamic alterations in coagulation pathways may facilitate earlier detection of vascular complications and inform personalized antithrombotic strategies.

## 1. Introduction

### Diabetes Mellitus and the Coagulation Cascade

Diabetes mellitus (DM) is a chronic metabolic disorder characterized by persistent hyperglycemia due to defects in insulin secretion, insulin action, or both. Its global prevalence continues to rise, with an estimated 463 million affected individuals in 2019, projected to reach 700 million by 2045 [1]. A significant proportion of patients remain undiagnosed, increasing the risk of both microvascular and macrovascular complications [2].

Among the most serious complications of diabetes is a heightened risk of thrombotic events and cardiovascular disease (CVD). Hyperglycemia, insulin resistance, and chronic low-grade inflammation contribute to a prothrombotic state through endothelial dysfunction, platelet hyperreactivity, increased coagulation factor levels, and impaired fibrinolysis [3,4,5]. Glycemic variability may further amplify thrombotic risk via oxidative stress and activation of inflammatory pathways, including NADPH oxidase and NF-κB signaling [6].

Coagulation abnormalities vary across diabetes subtypes. Type 1 diabetes (T1DM) is associated with absolute insulin deficiency and reduced nitric oxide bioavailability, promoting endothelial dysfunction. Type 2 diabetes (T2DM) involves insulin resistance and chronic inflammation, leading to elevated procoagulant factors and fibrinolytic inhibitors such as PAI-1. Gestational diabetes (GDM) typically presents with increased fibrinogen and reduced anticoagulant activity, predisposing to gestational thrombosis [4,7,8]. Other forms, including monogenic diabetes (e.g., MODY) and secondary diabetes, display coagulation alterations depending on their etiology [9]. Beyond T1DM, T2DM, and GDM, additional forms of diabetes include monogenic diabetes (e.g., maturity-onset diabetes of the young, MODY) and secondary diabetes resulting from endocrinopathies, pancreatic disorders, or medications. Certain MODY variants exhibit minimal coagulation abnormalities because endogenous insulin secretion is largely preserved and autoimmune inflammation is absent. Unlike T1DM, absolute insulin deficiency and immune-driven endothelial injury are uncommon in MODY, resulting in lower oxidative stress, reduced endothelial activation, and limited disruption of coagulation and fibrinolytic pathways [8,10].

Given the well-established relationship between diabetes and coagulation disorders, a detailed understanding of the specific alterations in coagulation markers is essential for both clinical risk assessment and the development of targeted therapies. Although there are numerous biomarkers involved in the pathogenesis of various forms of diabetes, this review aims to examine key coagulation biomarkers—including D-dimer, fibrinogen, prothrombin, tissue thromboplastin or tissue factor (TF), P-selectin, soluble urokinase plasminogen activator receptor (suPAR), Thrombomodulin (TM), Plasminogen activator inhibitor-1 (PAI-1), von Willebrand factor (vWF), and β-thromboglobulin (β-TG)—across various types of diabetes. By synthesizing current evidence, we aim to clarify the pathophysiological mechanisms underlying coagulation abnormalities in various forms of diabetes and explore their clinical implications for disease management and therapeutic strategies.

This review is structured as follows: We first provide a detailed overview of the pathophysiological mechanisms underlying these abnormalities to establish a foundation for understanding diabetic alterations. Next, we examine the changes in key coagulation biomarkers in various types of diabetes, including D-dimer, fibrinogen, prothrombin, TF, P-selectin, suPAR, TM, PAI-1, vWF, and β-TG. Finally, we discuss the clinical implications for disease management and therapeutic strategies.

## 2. Literature Search Strategy

This review was conducted as a narrative synthesis aimed at summarizing key hemostatic abnormalities in DM. The literature search was performed in PubMed, Scopus, and Web of Science using predefined keyword combinations and covered studies published between 2008 and 2026.

As this is not a systematic review, no formal protocol was registered, and PRISMA flow, dual screening, or structured risk-of-bias assessment were not applied. Studies were selected based on relevance and their contribution to the pathophysiological and clinical framework of the topic.

The selection of coagulation and platelet-related biomarkers was guided by three criteria: (i) biological relevance to key pathophysiological mechanisms in diabetes, including endothelial dysfunction, inflammation, and impaired fibrinolysis; (ii) repeated evaluation in observational and therapeutic studies across different diabetes phenotypes; and (iii) applicability to routine or near-routine clinical laboratory settings. Consequently, advanced functional assays such as thrombin generation testing, platelet function assays, and neutrophil extracellular trap (NET)-associated markers were not included, as their methodologies remain poorly standardized, their clinical thresholds are not well defined, and their use is largely confined to specialized research settings.

## 3. Pathophysiological Mechanisms of Coagulation Abnormalities in Various Forms of Diabetes

Under normal conditions, nonactivated platelets (NP) adhere to the damaged endothelium via VWF and collagen and subsequently undergo activation (1). Activated platelets (AP) provide a surface that supports the conversion of prothrombin to thrombin (2). Thrombin generation from prothrombin (3) leads to the transformation of soluble fibrinogen into fibrin, forming a thrombus (4). Thrombin further amplifies platelet activation through thrombin receptor signaling (5). The fibrinolytic pathway is also included. Endothelial cells continuously release tissue-type plasminogen activator (t-PA) (6) and PAI-1 (7). T-PA converts plasminogen into plasmin, enabling fibrin degradation (8), unless inhibited by PAI-1. Plasmin activity is rapidly neutralized by α_2_-plasmin inhibitor, resulting in the formation of the plasmin–α_2_-plasmin inhibitor complex (PIC) (9), the whole mechanism is known as endothelium–platelet–coagulation–fibrinolysis system [11].

**Figure 1:**
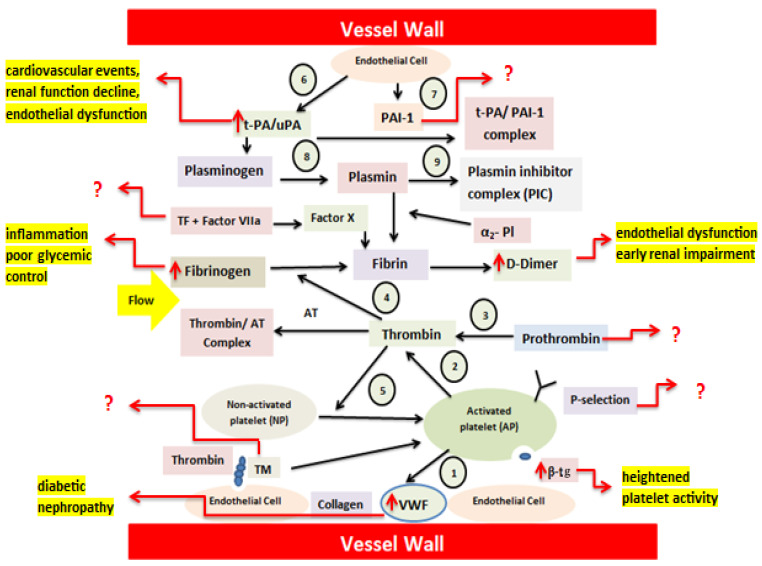
Schematic overview of coagulation abnormalities in Type 1 diabetes demonstrating dysregulation of the endothelium–platelet–coagulation–fibrinolysis system. Increased levels of D-Dimer, B-TG, vWF, Fibrinogen, uPAR contribute to the endothelial dysfunction, platelet hyperactivity, inflammation, and renal impairment in diabetes type 1 patients. Abbreviations: thromboplastin or tissue factor (TF), soluble urokinase plasminogen activator receptor (suPAR), Thrombomodulin (TM), Plasminogen Activator inhibitor-1 (PAI-1), von Willebrand factor (vWF), and β-thromboglobulin (β-TG). Arrows show changes or interactions (↑ increase, ↓ decrease); (?) indicates unclear or hypothetical mechanisms.Source: designed by authors with help of articles.

According to the pathophysiological mechanism of T1DM, chronic hyperglycemia–induced endothelial dysfunction enhances vWF release and platelet activation, leading to increased P-selectin expression and platelet aggregation. Activation of the tissue factor–dependent coagulation pathway promotes thrombin generation and fibrin formation, while alterations in fibrinolytic balance, reflected by changes in t-PA–mediated plasmin generation and fibrin turnover, contribute to a prothrombotic milieu. The combined effects of platelet hyperreactivity, excessive thrombin generation, and defective fibrin clearance create a prothrombotic state associated with vascular complications and early renal impairment in T1DM (Figure 1).

**Figure 2:**
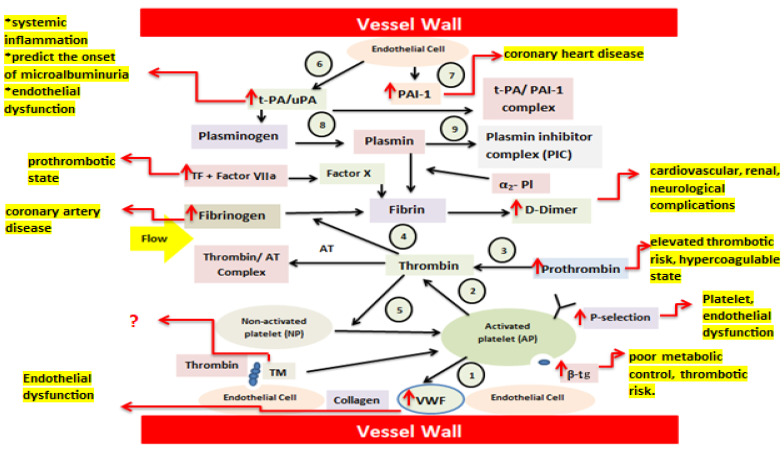
Schematic representation of coagulation abnormalities in Type 2 diabetes highlighting dysregulation of the endothelium–platelet–coagulation–fibrinolysis axis. Increased levels of D-Dimer, prothrombin, P-selectin, B-TG, vWF, TF, Fibrinogen, uPAR, and PAI contribute to the endothelial dysfunction, platelet dysfunction, prothrombotic state, systemic inflammation, renal impairment, coronary artery disease and neurological complications in type 2 diabetes patients. Abbreviations: thromboplastin or tissue factor (TF), soluble urokinase plasminogen activator receptor (suPAR), Thrombomodulin (TM), Plasminogen Activator inhibitor-1 (PAI-1), von Willebrand factor (vWF), and β-thromboglobulin (β-TG). Arrows denote directional changes (↑ increase, ↓ decrease) and biochemical interactions; (?) highlights mechanisms that remain uncertain or insufficiently evidenced.Source: designed by authors with help of articles.

In case of T2DM pathophysiology, insulin resistance, chronic hyperglycemia, and systemic inflammation induce endothelial dysfunction, resulting in increased release of vWF and enhanced platelet activation with elevated P-selectin expression. Activation of the tissue factor–dependent coagulation pathway promotes excessive thrombin generation and fibrin formation, accompanied by increased fibrinogen levels. Concurrent upregulation of PAI-1 and formation of t-PA/PAI-1 complexes impair fibrinolysis, leading to reduced plasmin activity despite increased fibrin turnover, as reflected by elevated D-dimer levels. The combined effects of platelet hyperreactivity, enhanced coagulation, and suppressed fibrinolysis create a hypercoagulable, prothrombotic state that contributes to cardiovascular, renal, and neurological complications in T2DM (Figure 2).

**Figure 3:**
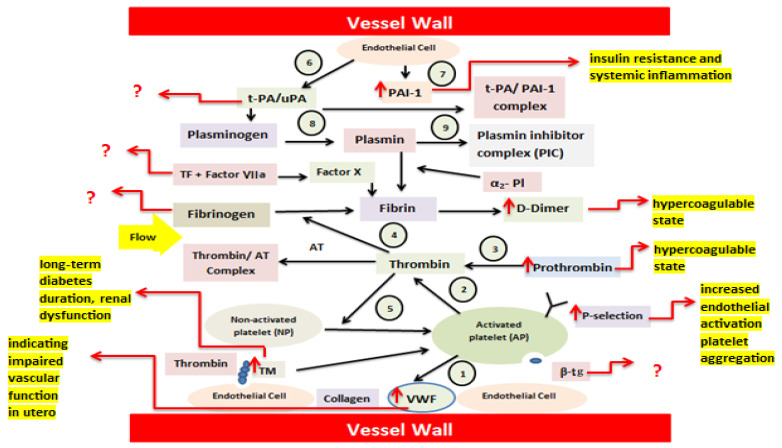
Schematic overview of coagulation abnormalities in gestational diabetes demonstrating dysregulation of the endothelium–platelet–coagulation–fibrinolysis system. Increased levels of D-Dimer, prothrombin, P-selectin, vWF, TM, and PAI contribute to the insulin resistance, endothelial activation, platelet aggregation, hypercoagulable state, renal dysfunction, and impaired vascular function in utero in the gestational diabetes patients. Abbreviations: thromboplastin or tissue factor (TF), soluble urokinase plasminogen activator receptor (suPAR), Thrombomodulin (TM), Plasminogen Activator inhibitor-1 (PAI-1), von Willebrand factor (vWF), and β-thromboglobulin (β-TG). Arrows indicate direction and magnitude of changes (↑ increase, ↓ decrease) or biochemical interactions, while question marks (?) denote hypothesized or unclear mechanisms where evidence is limited or not well established. Source: designed by authors with help of articles.

In the pathophysiology of gestational diabetes, pregnancy-related insulin resistance and inflammation cause endothelial activation with increased vWF (↑vWF) and platelet activation, reflected by elevated P-selectin (↑P-selectin). Enhanced tissue factor–driven coagulation increases prothrombin and thrombin generation, leading to fibrin formation and elevated D-dimer levels (↑D-dimer). Concurrent PAI-1–mediated suppression of fibrinolysis promotes a hypercoagulable state associated with adverse vascular risk (Figure 3).

**Figure 4:**
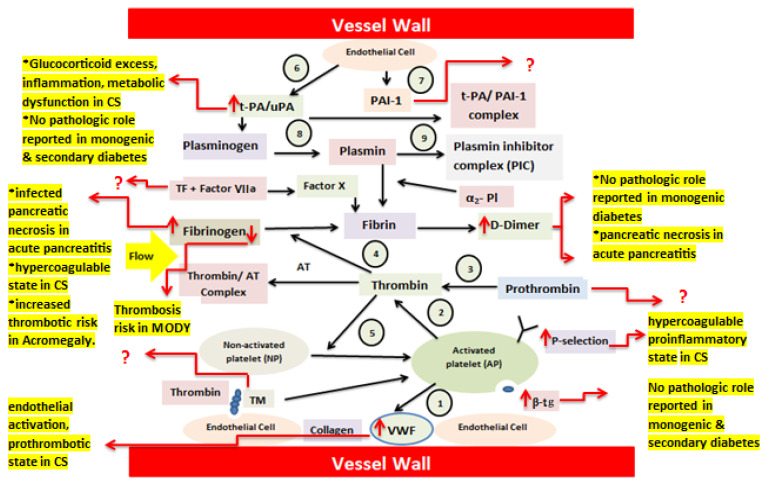
Schematic representation of the pathophysiological mechanisms underlying coagulation abnormalities in monogenic and secondary diabetes, illustrating biomarker-specific alterations within the endothelium–platelet–coagulation–fibrinolysis system. Increased levels of D-Dimer, P-selectin, vWF, and uPAR contribute to the various pathologies in secondary diabetes patients. Abbreviations: thromboplastin or tissue factor (TF), soluble urokinase plasminogen activator receptor (suPAR), Thrombomodulin (TM), Plasminogen Activator inhibitor-1 (PAI-1), von Willebrand factor (vWF), and β-thromboglobulin (β-TG). Arrows represent changes (↑ increase, ↓ decrease) or interactions, whereas (?) indicates unclear or not well-established mechanisms. Source: designed by authors with help of articles.

In monogenic and secondary diabetes pathophysiology, chronic hyperglycaemia, metabolic dysfunction, inflammation, and glucocorticoid excess in selected secondary forms drive endothelial activation, resulting in increased release of vWF (↑vWF) and enhanced platelet adhesion. Platelet activation is further supported by elevated P-selectin (↑P-selectin) and β-thromboglobulin, promoting platelet aggregation and platelet endothelium interactions. Activation of tissue factor-dependent coagulation pathways leads to increased prothrombin availability (↑prothrombin), augmented thrombin generation, and enhanced conversion of fibrinogen to fibrin. Accelerated fibrin turnover is reflected by increased D-dimer levels (↑D-dimer), indicating a shift toward a hypercoagulable state. Simultaneously, upregulation of plasminogen activator inhibitor-1 (↑PAI-1) and increased formation of t-PA/PAI-1 complexes limit effective plasmin generation despite preserved or increased t-PA/u-PA activity, resulting in impaired fibrinolysis. Collectively, endothelial dysfunction, platelet hyperreactivity, enhanced coagulation, and attenuated fibrinolytic capacity contribute to a sustained prothrombotic milieu in monogenic and secondary diabetes, potentially increasing the risk of thrombosis and vascular complications (Figure 4).

## 4. Role of Major Coagulation Biomarkers in the Pathogenesis of Diabetes Subtypes

### 4.1. D-Dimer

D-dimers are protein dimers made up of two fibrin D fragments connected by a cross-link. This little protein fragment is released into circulation after fibrinolysis breaks down a blood clot. The plasmin enzyme breaks down cross-linked fibrin to create D-dimer. The production of fibrin and its subsequent breakdown by the body’s natural fibrinolytic system affect the levels of D-dimer in the plasma, which act as a coagulation indicator [12]. D-dimer, a fibrin degradation product, is a key biomarker of coagulation activation and is widely used to assess thrombotic risk. Elevated D-dimer levels have been reported across multiple types of diabetes, often correlating with vascular complications, inflammation, and organ dysfunction [12].

#### 4.1.1. T1DM

In patients with T1DM, elevated D-dimer levels are primarily linked to endothelial dysfunction and early renal impairment. Domingueti et al. demonstrated that D-dimer levels increase with declining renal function, suggesting an association with early diabetic kidney disease (DKD) [13]. However, the cross-sectional nature of the study and lack of control for confounding factors such as inflammation or medication use limit causal interpretation. El Asrar et al. also reported a positive correlation between D-dimer and albumin-to-creatinine ratio (ACR), although levels were generally lower in T1DM than in T2DM patients [14]. D-dimer reflects downstream fibrin turnover and vascular injury and therefore lacks sufficient specificity to differentiate inflammatory- from metabolically driven nephropathy in T1DM. Collectively, these findings support the potential utility of D-dimer as a biomarker of microvascular complications in T1DM, particularly nephropathy, though standardized clinical thresholds remain undefined.

#### 4.1.2. T2DM

In T2DM, D-dimer elevation has been implicated in cardiovascular, renal, and neurological complications. Zhou et al. found that elevated D-dimer levels independently predicted adverse cardiovascular outcomes in T2DM patients with ACS [15]. Other studies have linked D-dimer to early kidney dysfunction [16], cognitive impairment in patients with carotid plaques [17], and peripheral neuropathy [18]. Fernández–Castañer et al. reported increased D-dimer levels even in prediabetic first-degree relatives, suggesting early coagulation activation before hyperglycemia onset [19]. However, small sample sizes and lack of longitudinal data limit generalizability. D-dimer has also been associated with increased risk in diabetic patients infected with COVID-19, especially when combined with postprandial hyperglycemia [20]. Despite its potential, variability in study design and lack of universal thresholds restrict its current clinical use in T2DM.

#### 4.1.3. GDM

Pregnancy is associated with a physiological hypercoagulable state, which appears to be exacerbated in GDM. Studies report significantly elevated D-dimer levels in GDM patients, particularly in the third trimester and postpartum period [21]. A study among Saudi women found that maternal age, gestational age, and GDM history were independently associated with increased D-dimer levels [22]. Mallah et al. observed that elevated D-dimer persisted up to 14 days after cesarean section in GDM patients, indicating a prolonged thrombotic risk [23]. However, most findings are based on cross-sectional analyses with limited adjustment for confounders such as BMI and comorbidities. Prospective studies are needed to determine whether D-dimer can serve as a reliable predictor of thrombotic complications in GDM.

#### 4.1.4. Monogenic and Secondary Diabetes

Although D-dimer has not been extensively studied in monogenic diabetes, data from secondary diabetes subtypes suggest disease-specific patterns. In patients with diabetes secondary to pancreatitis, elevated D-dimer levels were found to predict pancreatic necrosis and overall disease severity in acute pancreatitis cohorts [24,25,26,27]. However, these studies did not stratify patients by diabetic status, limiting conclusions about diabetes-specific effects. In Cushing’s syndrome-associated diabetes, D-dimer levels ≥2.6 μg/mL were strong predictors of venous thromboembolism, likely reflecting cortisol-induced hypercoagulability [28]. The relationship between D-dimer, insulin resistance, and glucose metabolism in these populations remains unclear and warrants further investigation.

#### 4.1.5. Diabetic Complications

Elevated D-dimer levels have been associated with the presence and severity of microvascular complications. In diabetic retinopathy (DR), Zhao et al. observed significantly higher D-dimer levels in patients with proliferative DR compared to nonproliferative forms [29]. In diabetic nephropathy (DN), Yu et al. found correlations between D-dimer and histopathological markers such as interstitial fibrosis, tubular atrophy, and reduced estimated glomerular filtration rate [30]. However, these findings are primarily from cross-sectional studies with potential selection bias, especially in biopsy-confirmed DN cohorts. Longitudinal studies are needed to determine whether D-dimer can serve as a predictive marker for the development or progression of microvascular complications.

### 4.2. Fibrinogen

Since its discovery over 350 years ago, fibrinogen has been the subject of intensive research that has shown its extraordinary properties. This huge, 340 kDa hexameric homodimer has a complicated structure that supports its many functions in homeostasis and hemostasis. Both constitutive (basal) secretions from the liver and inducible upregulation in response to inflammatory events are involved in the control of fibrinogen production at the levels of transcription and translation. Additionally, alternative splicing contributes to the biochemistry of coagulation by producing fibrinogen variants with unique characteristics [12].

Fibrinogen is a major acute-phase reactant and a critical component of the coagulation cascade. Hyperfibrinogenemia has been implicated in the pathogenesis of thromboembolic events and vascular complications in various forms of DM. Elevated levels of fibrinogen not only reflect systemic inflammation but may also actively contribute to atherogenesis, endothelial dysfunction, and microvascular damage.

#### 4.2.1. T1DM

In T1DM, fibrinogen levels are generally lower than in T2DM but still contribute to vascular risk. Comparative studies have consistently shown significantly higher fibrinogen levels in T2DM compared to T1DM, correlating with inflammation and poor glycemic control [13,14]. However, the absence of longitudinal data limits the ability to establish causality. While elevated fibrinogen may serve as a marker for atherothrombotic risk, current evidence is insufficient to support its routine use in T1DM for risk prediction or therapeutic guidance.

#### 4.2.2. T2DM

In T2DM, elevated fibrinogen is a consistent finding and is independently associated with increased cardiovascular risk. Gupta et al. reported significantly elevated fibrinogen levels in T2DM patients, independent of the presence of coronary artery disease [31]. Bembde found a strong positive correlation between fibrinogen and glycated hemoglobin (HbA1c), suggesting that poor glycemic control amplifies fibrinogen elevation [32]. Barazzoni et al. proposed that hyperfibrinogenemia may precede cardiovascular complications, regardless of albuminuria status [33]. Additional studies by Razak and Sultan further linked fibrinogen to diabetes duration and lipid abnormalities [34]. However, variability in methodologies and lack of standardized thresholds limit the clinical applicability of these findings. Despite its promise, prospective trials are needed to determine whether fibrinogen reduction strategies can mitigate cardiovascular outcomes in T2DM.

#### 4.2.3. Monogenic Diabetes (e.g., MODY)

In monogenic diabetes, data on fibrinogen are limited. Jones and Peterson investigated fibrinogen kinetics in adults with diabetes and observed that while aspirin and dipyridamole had no effect, heparin normalized fibrinogen survival, implicating thrombin-mediated mechanisms [35]. Sagar et al. compared fibrin network properties among HNF1A-MODY, T1DM, and T2DM patients, finding that MODY patients displayed less thrombogenic fibrin structures than those with T2DM, possibly due to lower C3 complement levels [36,37]. Although suggestive of a lower thrombotic risk in MODY, the findings require validation using functional clot assays and mechanistic studies.

#### 4.2.4. Secondary Diabetes

##### Pancreatitis-Associated Diabetes

Fibrinogen appears elevated in individuals with diabetes secondary to chronic pancreatitis (CP-DM), suggesting a stronger prothrombotic tendency than in T1DM [38]. Additionally, a study in patients with acute pancreatitis found that fibrinogen-like protein 1 (FGL-1) correlated with infected pancreatic necrosis (IPN), although the diabetic status of participants was not stratified [39]. The dated methodologies and lack of diabetes-specific analysis limit the conclusions.

##### Cushing’s Syndrome

In Cushing’s syndrome (CS), multiple studies have demonstrated elevated fibrinogen levels, reflecting a hypercoagulable state induced by chronic hypercortisolism [40,41]. These increases may contribute to vascular complications in patients with Cushing’s-related diabetes, but direct links to glucose metabolism and long-term vascular outcomes remain insufficiently studied.

##### Acromegaly

Fibrinogen levels are consistently elevated in acromegalic patients and may be linked to increased thrombotic risk. Landin-Wilhelmsen et al. reported significantly higher fibrinogen levels in acromegaly and a positive correlation with IGF-I levels [42]. Kyriakakis et al. found denser clot structures in acromegalic patients, suggesting increased fibrin-related thrombogenicity [43]. Sartorio et al. observed hyperfibrinogenemia independent of IGF-I levels, suggesting that acromegaly itself may drive coagulation abnormalities [44]. However, none of these studies specifically evaluated fibrinogen levels in diabetic subgroups, which limits their direct relevance to secondary diabetes.

#### 4.2.5. Diabetic Complications

Elevated fibrinogen levels have also been associated with diabetic peripheral neuropathy (DPN). One study found that changes in fibrinogen function, measured via the K value and angle α, were strongly correlated with DPN diagnosis [45]. However, the small sample size and lack of a control group limit the study’s broader applicability. Further research is needed to determine whether fibrinogen alterations directly contribute to neuropathic mechanisms or serve merely as markers of systemic inflammation.

### 4.3. P-Selectin

P-selectin plays a major part in the interaction between blood cells. This specific protein, which weighs 140 Da, is an essential membrane glycoprotein that helps activated platelets and endothelial cells adhere to neutrophils and monocytes. P-selectin is produced by endothelial cells and stored in Weibel-Palade bodies, which are specialized granules. Alpha granules contain P-selectin in platelets. Leukocytes begin to migrate towards inflammatory endothelium when P-selectin attaches to its matching ligand, P-selectin glycoprotein ligand one (PSGL-1), on leukocytes. This is the initial phase of drawing leukocytes to inflammatory areas [12]. P-selectin is an adhesion molecule expressed on activated endothelial cells and platelets, playing a central role in leukocyte recruitment and thrombus formation. Elevated levels of soluble P-selectin (sP-selectin) are consistently observed in diabetes and are associated with platelet hyperactivity and endothelial dysfunction, contributing to vascular complications.

#### 4.3.1. T1DM

Jilma et al. [46] reported elevated circulating P-selectin levels in T1DM patients receiving intensified insulin therapy, suggesting enhanced platelet activation. However, their study had a small sample size and did not adjust for confounders such as lipid profile or inflammatory markers, limiting its generalizability. Despite these limitations, P-selectin remains a potential therapeutic target in T1DM, warranting confirmation in larger, controlled studies.

#### 4.3.2. T2DM

Numerous studies have documented increased sP-selectin levels in T2DM, correlating with fasting plasma glucose, hypertriglyceridemia, and vWF, indicating a role in both platelet and endothelial dysfunction [47,48]. However, the lack of correlation with platelet factor 4 (PF4) raises questions about its specificity. Animal models have shown that P-selectin may not be the primary mediator of myocardial injury in diabetic ischemia–reperfusion, challenging its centrality in T2DM cardiovascular pathology [49].

Ali et al. [50] demonstrated that sP-selectin levels in T2DM correlated with inflammatory and metabolic markers, possibly outperforming high-sensitivity C-reactive protein (hsCRP) in predicting coronary events. Still, the small cohort and cross-sectional design limit its predictive utility. Elevated P-selectin levels have also been observed in acute coronary syndromes (e.g., myocardial infarction and unstable angina), suggesting a role in identifying high-risk individuals [51].

Post-hypoglycemic impairment of platelet response to prostacyclin (PGI2) and reduced inhibition of P-selectin expression have been observed, linking hypoglycemia to enhanced thrombotic risk [52]. However, the absence of data on antiplatelet use weakens these findings.

Genetic studies have explored polymorphisms in the SELP gene. The 290Asn (S290N) variant has been linked to albuminuria [53], while the Thr715Pro polymorphism showed no significant cardiovascular risk association [54]. Kaur et al. [55] further implicated SELP noncoding variants in arterial stiffness and sP-selectin levels. While suggestive, these findings require validation in larger, multiethnic populations with consistent endpoints.

#### 4.3.3. GDM

Elevated P-selectin levels have been reported in GDM, reflecting increased endothelial activation and platelet aggregation [56]. However, most studies do not adequately adjust for confounding factors such as obesity and insulin resistance, which independently affect endothelial function. Additionally, methodological inconsistencies in measuring P-selectin limit comparability. Future studies should control for these variables to clarify whether P-selectin is a GDM-specific biomarker.

#### 4.3.4. Secondary Diabetes

##### Pancreatitis-Associated Diabetes

In murine models of pancreatitis, anti-P-selectin antibodies have been shown to reduce pancreatic inflammation, suggesting a mechanistic link between P-selectin and thrombosis in pancreatitis. However, there is a lack of clinical studies evaluating P-selectin in pancreatitis-related diabetes, making its relevance uncertain.

##### Cushing’s Syndrome

Patients with CS, particularly those with concurrent diabetes and hypertension, exhibit elevated P-selectin levels, reflecting a hypercoagulable and proinflammatory state [57]. This finding aligns with the increased cardiovascular burden in CS-associated secondary diabetes but requires longitudinal validation.

#### 4.3.5. Diabetic Complications

P-selectin expression has been linked to DR and nephropathy. In DR, upregulation was observed in choroidal—but not retinal—vessels, with no detectable E-selectin in retinal tissues [58]. This suggests tissue-specific expression patterns, although inconsistent detection techniques limit reproducibility. In dialysis-dependent DN, elevated levels of sP-selectin, VCAM-1, and ICAM-1 indicate ongoing endothelial activation [59]. However, the cross-sectional design precludes conclusions about causality. Longitudinal studies are needed to determine whether P-selectin can serve as a predictor of microvascular complication progression.

### 4.4. suPAR (Soluble Urokinase Plasminogen Activator Receptor)

Soluble Urokinase Plasminogen Activator Receptor (suPAR) is a broadly distributed, non-specific biomarker of inflammation that is generated in response to inflammatory stimuli, regardless of the underlying aetiology. It is a soluble type of urokinase receptor, a cell membrane receptor for urokinase-type plasminogen activator that is selective to glycosylphosphatidylinositol (GPI) anchors. Urokinase transforms plasminogen into plasmin in order to break up blood vessel thrombi. The cascade of fibrinolysis is therefore started. Leukocyte migration into tissue is thought to be facilitated by the chemotactic function of SuPAR, a 60 kDa glycoprotein that is created when the GPI anchor is destroyed. It also contributes to tissue remodeling after damage. The molecule being measured is the membrane-bound receptor in its active form, which is expressed in a variety of cell types, including white blood cells, fibroblasts, tissue macrophages, renal tubular cells, and endothelial cells [12].

uPAR is a stable plasma biomarker reflecting immune activation, systemic inflammation, and endothelial dysfunction. Its role in diabetes pathogenesis and complications has been increasingly recognized across various diabetes subtypes.

#### 4.4.1. T1DM

suPAR was identified as an independent predictor of cardiovascular events, renal function decline, and all-cause mortality in T1DM patients. Although the study adjusted for traditional risk factors, its observational design limits causal inference. Moreover, standardized cutoff thresholds for clinical decision-making are currently lacking, and further prospective interventional studies are warranted [60,61].

#### 4.4.2. T2DM

suPAR levels have been shown to correlate with albuminuria and may predict the onset of microalbuminuria, potentially allowing earlier risk stratification than albuminuria alone [62]. However, Rotbain et al. [63] found that suPAR did not predict the therapeutic response to dapagliflozin, suggesting its limited utility in guiding treatment selection.

Despite being a recognized marker of immune activation, the role of suPAR in T2DM development appears complex. Some studies suggest a stronger predictive value in overweight individuals and nonsmokers [64], but heterogeneity in study design, suPAR quantification methods, and patient characteristics limits cross-study comparisons.

Persistent immune activation may underlie sustained elevation of inflammatory–coagulation markers such as suPAR. suPAR reflects chronic immune system activation and macrophage-driven inflammation and is relatively insensitive to short-term metabolic improvement. Even after glucose-lowering therapy or vascular revascularization, structural endothelial damage, ongoing tissue remodeling, and immune memory may sustain low-grade inflammation, thereby maintaining a prothrombotic milieu despite apparent metabolic control. Persistent elevation of suPAR despite revascularization or conventional glucose-lowering therapy implies that residual immune activation may drive ongoing cardiovascular risk in T2DM patients [65]. Magnus and Mona [66] reported higher suPAR levels in T2DM than in T1DM, suggesting more pronounced systemic inflammation. However, a lack of longitudinal data hampers understanding of its role in disease progression.

Haugaard et al. [67] demonstrated an independent association between suPAR and incident T2DM in nonsmokers. Interestingly, this relationship was absent in smokers, possibly due to competing risk factors such as smoking-induced inflammation. Additionally, elevated suPAR levels have been observed in T2DM patients with cardiovascular disease, though it remains unclear whether this association is independent of classical cardiovascular risk factors [68].

#### 4.4.3. GDM

Higher suPAR levels have been reported in pregnant women who later developed GDM, with one model demonstrating 96.6% specificity and 97.5% sensitivity when suPAR was combined with other biomarkers [69]. However, this study lacked external validation and was conducted in a demographically homogeneous population. Prospective multicenter studies are needed to confirm these findings across diverse populations before incorporating suPAR into GDM screening algorithms.

#### 4.4.4. Secondary Diabetes

Evidence on coagulation abnormalities in secondary diabetes, particularly in acromegaly and pancreatogenic diabetes, remains limited and is frequently derived from studies in which diabetic status was not consistently stratified. This limits the ability to directly attribute observed coagulation changes to hyperglycemia alone. However, available data suggest that disease-specific inflammatory and endocrine mechanisms may contribute to coagulation dysregulation independent of glycemic control.

##### Pancreatitis-Associated Diabetes

suPAR has been implicated in pancreatic fibrosis in chronic pancreatitis [70], suggesting a role in disease progression. However, no studies have yet evaluated suPAR specifically in pancreatitis-related diabetes. Additionally, there is a lack of data on other coagulation-related markers (e.g., thrombomodulin, β-TG, vWF, P-selectin, PAI-1) in diabetic pancreatitis. Future studies should aim to stratify biomarker expression between diabetic and nondiabetic pancreatitis patients and explore whether coagulation-targeted therapies offer clinical benefit.

##### Cushing’s Syndrome

Despite the known prothrombotic state in CS, no studies have evaluated suPAR in patients with CS or CS-related diabetes. Investigating suPAR in this context may help elucidate the interplay between glucocorticoid excess, inflammation, and metabolic dysfunction. Standardized methodologies and longitudinal studies are needed to determine whether suPAR plays a role in CS-associated hypercoagulability.

#### 4.4.5. Diabetic Complications

suPAR has been linked to the severity and duration of DN, with higher levels associated with advanced pathological class and impaired renal function [71]. However, variability in patient demographics and therapeutic regimens across studies may confound these associations. Aslan et al. [72] also identified suPAR as a potential marker for diabetic foot infections, though its specificity remains uncertain. Larger, well-controlled studies are necessary to validate suPAR’s role across diabetes-related complications, particularly in distinguishing inflammation-driven versus metabolic pathology.

### 4.5. Tissue Plasminogen Activator (tPA)

One serine protease that is essential to fibrinolysis is tissue-type plasminogen activator (tPA). Its main function is to convert plasminogen into plasmin, which breaks down fibrin clots. Hepatocytes and endothelial cells release tPA into the circulation. Furthermore, tPA is also produced by immune cells such as monocytes and T cells. Apart from its role in hemostasis, tPA is also involved in the control of several inflammatory illnesses and functions as a powerful regulator of inflammation [12].

#### Type 1 Diabetes Mellitus (T1DM)

Myrup et al. [73] observed elevated tPA activity in T1DM patients without nephropathy, suggesting early alterations in the fibrinolytic system. This increase may reflect a compensatory response to endothelial dysfunction in the absence of advanced microvascular complications. However, animal studies have shown that chronic hyperglycemia suppresses tPA expression, thereby exacerbating ischemic injury [74]. These divergent findings underscore the complexity of fibrinolytic regulation in T1DM and indicate the need for further mechanistic and longitudinal studies to clarify tPA’s role in diabetes-related thrombosis.

### 4.6. vWF

In relation to hemostasis, the vWF protein carries out three essential activities. In order to prolong its life in the blood, it first attaches itself to factor VIII (FVIII). Second, it connects itself to collagen in the subendothelial matrix. Finally, it attaches itself to platelets and acts as a link between them and the subendothelial matrix. As a result, vWF is essential for attracting platelets to the damage site. The unique bleeding symptoms seen in vWD are explained by the absence of vWF, which highlights its crucial role in primary and secondary hemostasis [12].

#### 4.6.1. T1DM

Elevated plasma vWF levels have been observed in children with T1DM prior to the onset of microalbuminuria, indicating a potential role as an early marker of diabetic nephropathy [75]. Additionally, increased levels of vWF, ADAMTS13 (a disintegrin and metalloproteinase with thrombospondin type 1 motif, member 13), and D-dimer have been reported in T1DM patients with declining renal function, supporting their association with endothelial dysfunction [15]. However, these findings are based on observational studies, limiting causal interpretation. Interventional trials are needed to determine whether modulating the vWF–ADAMTS13 axis can improve renal outcomes. Interestingly, similar patterns have been reported in T2DM, with increased vWF and reduced ADAMTS13 activity in patients with nephropathy, further implicating endothelial dysfunction in disease progression [76].

#### 4.6.2. T2DM

In T2DM, an imbalance between elevated vWF and its cleaving protease ADAMTS13 exacerbates prothrombotic tendencies by promoting the accumulation of ultra-large vWF multimers on the endothelial surface. This effect synergizes with elevated plasminogen activator inhibitor-1 (PAI-1), which inhibits tissue plasminogen activator and suppresses fibrinolysis. Consequently, the vWF/ADAMTS13 imbalance amplifies the fibrinolytic impairment caused by PAI-1, creating a self-reinforcing prothrombotic environment. Such interactions highlight the need to consider not only absolute biomarker levels but also the functional interplay between coagulation and fibrinolytic pathways when evaluating thrombotic risk in T2DM [77].

In T2DM, vWF is consistently associated with chronic vascular complications. A recent meta-analysis confirmed significantly higher vWF levels in T2DM patients with cardiovascular disease (CVD) compared to those without CVD, supporting its prognostic value [77]. Nevertheless, heterogeneity in measurement techniques and patient characteristics across studies complicates interpretation. Endothelial dysfunction in T2DM is reflected by elevated vWF and urinary albumin levels, although the weak correlation between them suggests distinct regulatory mechanisms [78]. Further mechanistic studies are required to clarify this relationship.

#### 4.6.3. GDM

Maternal hyperglycemia has been associated with fetal endothelial dysfunction. A study in GDM pregnancies reported significantly elevated fetal vWF levels and decreased aortic propagation velocity (APV), indicating impaired vascular function in utero [79]. The inverse correlation between APV and vWF suggests a potential link between maternal metabolic status and offspring cardiovascular risk. However, longitudinal studies are necessary to determine whether vWF can serve as a predictive marker of long-term cardiovascular outcomes in these children.

#### 4.6.4. Secondary Diabetes

##### Cushing’s Syndrome-Related Diabetes

Increased vWF antigen levels have been reported in patients with CS, particularly in ACTH-dependent subtypes, suggesting endothelial activation and a prothrombotic state [39,80]. This supports the hypothesis that cortisol-induced endothelial dysfunction contributes to vascular complications in secondary diabetes.

##### Acromegaly-Related Diabetes

Although vWF has not been specifically measured in diabetic patients with acromegaly, hypercoagulability has been reported in this population. Campello et al. [81] found increased levels of fibrinogen, factor VIII, and thrombin generation in acromegalic patients, but did not assess vWF directly. Whether vWF elevation in this context is driven by excess GH/IGF-1 or by coexistent diabetes remains unknown, and future studies are warranted to dissect these contributions.

#### 4.6.5. Diabetes-Related Complications

Endothelial dysfunction marked by elevated vWF levels is implicated in the pathogenesis of DN. Stehouwer [82] demonstrated that increased vWF was associated with DN progression, although the study did not adjust for glycemic variability. Animal studies have shown that ADAMTS13 deficiency worsens diabetic renal injury, whereas vWF deficiency may be protective, suggesting a pathogenic role for the vWF/ADAMTS13 imbalance [83]. Validation in human studies is required before clinical application.

### 4.7. β-Thromboglobulin (β-TG)

Beta-thromboglobulin (β-TG) is a chemokine protein secreted by platelets. The protein β-TG is made up of four subunits, each of which has eight amino acids and a molecular weight of around 35,000. It has a connection to platelet factor 4. The antigenic elements and amino acid structures of the two proteins are comparable. A new NH2 terminal that is similar to the NH2 terminal of BTG is created when the terminal four amino acids in the NH2 terminal of platelet factor 4 are removed. It has been proposed that BTG is produced by proteolytic activity from platelet factor 4 [84].

#### 4.7.1. T1DM

Elevated β-TG levels have been reported in T1DM patients, indicating heightened platelet activity even in individuals with good glycemic control [85,86]. However, short-term interventions aimed at improving glucose levels did not significantly alter hemostatic abnormalities, suggesting that platelet hyperactivity may persist despite transient improvements in glycemia [87]. This raises important questions about the long-term efficacy of glycemic management in reversing prothrombotic tendencies in T1DM.

#### 4.7.2. T2DM

β-TG, a marker of platelet degranulation, and fibrinopeptide A (FpA), a marker of fibrin formation, have been shown to be elevated in T2DM patients, particularly in those with vascular complications. A study involving 68 non-insulin-dependent diabetic patients demonstrated significantly increased plasma β-TG and FpA levels in individuals with either microvascular or macrovascular complications [88]. These elevations were accompanied by higher HbA1c and blood pressure levels, underscoring the interplay between poor metabolic control and thrombotic risk. However, the cross-sectional design and limited sample size of this study restrict its ability to determine causality. Further prospective studies are warranted to explore whether β-TG and FpA serve as predictive biomarkers for the development of diabetic complications. Additionally, confounding variables such as lipid profiles and systemic inflammation were not fully accounted for, limiting the generalizability of the findings.

#### 4.7.3. Diabetes-Related Complications

Increased β-TG levels have been associated with diabetic microangiopathy, reflecting enhanced platelet activation [89]. Interestingly, improved glycemic control has been shown to reduce β-TG levels, suggesting a potentially reversible process. However, β-TG concentrations did not correlate significantly with the presence or severity of diabetic retinopathy, nephropathy, or macrovascular disease in some studies [90]. These inconsistencies highlight the need for additional research to clarify the specificity and clinical utility of β-TG as a biomarker for diabetes-related vascular complications.

### 4.8. Prothrombin

Coagulation factor II, or prothrombin, is an essential component of blood coagulation. Prothrombin is converted into its active form, thrombin (factor IIa), via enzymatic cleavage. The conversion of fibrinogen into fibrin, which is necessary for the development of blood clots, is catalyzed by thrombin. Prothrombin therefore guarantees the clot formation process [40].

#### 4.8.1. T2DM

Alterations in the coagulation cascade are characteristic of T2DM and contribute to the elevated thrombotic risk observed in these patients. Several studies have investigated prothrombin and associated parameters, yet their findings remain inconsistent. For example, some research has reported prolonged prothrombin time (PT) and activated partial thromboplastin time (APTT) in T2DM patients, suggesting a paradoxical risk for bleeding [91]. In contrast, other studies have found shortened PT and APTT values, coupled with increased platelet counts, indicating a hypercoagulable state and enhanced thrombotic potential [92,93]. These discrepancies may be attributed to differences in glycemic control, patient demographics, comorbid conditions, and assay methodologies. To clarify the role of prothrombin in T2DM-related coagulopathy, standardized, large-scale, prospective studies are warranted.

#### 4.8.2. GDM

Data on prothrombin activity in GDM are limited and inconclusive. One study conducted in Bangladesh found no significant differences in PT or APTT values between GDM and non-GDM groups [94]. However, the study’s small sample size and lack of statistical power limit the reliability of its findings. Conversely, other reports suggest that GDM may be associated with decreased PT and APTT, along with increased levels of fibrinogen and plasminogen, indicating a hypercoagulable state [8]. Notably, few studies have accounted for physiological changes in coagulation across different trimesters or the postpartum period, which may contribute to variability in results. Longitudinal studies are needed to elucidate the temporal evolution of prothrombin activity during and after pregnancy in GDM patients.

#### 4.8.3. Cushing’s Syndrome-Related Diabetes

Patients with CS have been shown to exhibit shortened PT and PTT, indicating increased thrombin generation and a heightened prothrombotic state [95]. Elevated tissue thromboplastin activity in ACTH-dependent CS further amplifies this risk [80]. In the context of CS-associated secondary diabetes, persistent hypercortisolism may exacerbate coagulation abnormalities, but specific data on prothrombin levels in diabetic CS patients remain scarce. Future research should aim to differentiate the direct effects of hypercortisolism from those attributable to hyperglycemia.

### 4.9. Tissue Thromboplastin

Tissue thromboplastin, also known as tissue factor/CD142, is a crucial protein that binds to and activates the plasma serine protease factor VIIa to start the blood coagulation cascade in response to vascular damage. Tissue factor is an essential part of hemostasis and is important in coagulation-related clinical disorders. It promotes the coagulation process in a number of thrombotic conditions, sepsis-related coagulopathies, and other types of disseminated intravascular coagulation. Beyond hemostasis, tissue factor has been shown to have further functions in cellular signaling, inflammatory processes, vasculogenesis, tumor development, and metastasis [96].

#### T2DM

Patients with T2DM have a 2–4-fold increased risk of thrombotic cardiovascular events, which may be partially attributed to abnormalities in tissue thromboplastin activity and related coagulation factors. Some studies have reported reduced APTT in T2DM patients, suggesting a hypercoagulable state that may be detectable even in early stages of the disease [97]. However, the diagnostic and prognostic value of APTT remains controversial, as it can be influenced by systemic inflammation and metabolic disturbances unrelated to diabetes.

Sapkota et al. [98] observed elevated fibrinogen levels and shortened APTTs in T2DM patients compared to healthy controls, supporting the hypothesis of increased thrombogenicity. Yet, the study lacked long-term follow-up to assess whether these abnormalities predict clinical outcomes. Furthermore, research investigating oxidative stress and coagulation dynamics found that increased superoxide dismutase (SOD) activity correlated with shortened APTT in T2DM patients, indicating that oxidative stress may contribute to hemostatic imbalance [99].

Vaidyula et al. [100] demonstrated that the combination of hyperinsulinemia and hyperglycemia upregulates tissue factor expression, further promoting a prothrombotic state. These findings emphasize the multifactorial nature of coagulation dysfunction in T2DM and highlight the need for mechanistic and longitudinal studies to clarify the clinical implications of tissue thromboplastin abnormalities in diabetic populations.

### 4.10. PAI-1

Plasminogen activator inhibitor-1 is a crucial regulator of the plasminogen/plasmin system and a member of the serine protease inhibitor (serpin) family. In this system, plasminogen activators (PAs) mediate the proteolytic cleavage that transforms the zymogen plasminogen into the active enzyme plasmin. Plasmin mainly contributes to fibrinolysis when it is mediated by tPA, which breaks down the insoluble fibrin meshwork that makes up blood clots. The plasminogen/plasmin system’s role extends to pericellular proteolysis linked to tissue remodeling and cell migration through urokinase-type PA (uPA)-mediated plasminogen activation [101].

#### 4.10.1. T2DM

Elevated PAI-1 levels are a hallmark of T2DM and are linked to impaired fibrinolysis, chronic inflammation, and cardiovascular risk. While platelets are a known source of circulating PAI-1, Mossberg et al. [102] found no significant differences in PAI-1 mRNA or protein expression in platelets from diabetic, obese, and lean individuals, suggesting a limited role for platelet-derived PAI-1. However, the dynamics of PAI-1 synthesis and secretion in different tissues remain incompletely understood.

Coudriet et al. [103] demonstrated that PAI-1 contributes to unresolved inflammation in obesity-related T2DM by inhibiting hepatocyte growth factor activation. Moreover, alterations in PAI-1’s circadian rhythm—especially in insulin-resistant patients with cardiovascular disease—may reflect hyperinsulinemia-driven atherogenesis [104].

Genetic and environmental factors also modulate PAI-1 expression. A study in Tunisian patients identified the −675 4G/5G polymorphism as a primary determinant of plasma PAI-1 levels, influenced secondarily by central obesity, dyslipidemia, and hyperglycemia [105]. However, inconsistent effect sizes across studies indicate the need for larger, well-controlled prospective cohorts to confirm causality [106].

Interestingly, while high PAI-1 is associated with increased coronary heart disease risk, it appears inversely related to diabetic retinopathy, suggesting context-dependent effects [107]. In adolescents with T2DM, elevated PAI-1 and reduced tPA activity point to impaired fibrinolysis as an independent cardiovascular risk factor, unaffected by acute dietary factors [108].

#### 4.10.2. GDM

Multiple studies have reported elevated PAI-1 levels in patients with GDM, with correlations to hyperglycemia and inflammatory mediators [109]. Nonetheless, there is debate regarding whether PAI-1 acts as an independent predictor of GDM or merely reflects insulin resistance and systemic inflammation. A meta-analysis highlighted significant heterogeneity among studies, underlining the necessity for standardized PAI-1 quantification protocols and stratification by disease severity [110].

The role of PAI-1 in postpartum thrombotic risk remains understudied. Given the thrombotic implications of PAI-1 overexpression, future research should explore its predictive utility in postpartum risk stratification among women with prior GDM.

#### 4.10.3. Secondary Diabetes

##### Cushing’s Syndrome-Related Diabetes

In patients with CS, elevated PAI-1 levels have been consistently reported and positively correlate with serum cortisol concentrations [41]. Since PAI-1 suppresses fibrinolysis, its increase in CS-related diabetes may contribute to both thrombosis and metabolic dysfunction. Further research is needed to clarify the mechanistic links between glucocorticoid excess, PAI-1 expression, and insulin resistance.

##### Acromegaly-Related Diabetes

The relationship between PAI-1 and acromegaly is complex. Sartorio et al. [44] found no significant increase in PAI-1 levels among acromegalic patients, contrasting with other studies that suggest a hypercoagulable state in this population. Conversely, Delaroudis et al. [111] reported a significant reduction in PAI-1 levels following partial disease control, along with improvements in glucose and lipid metabolism. While these results suggest that PAI-1 may contribute to thrombotic risk in acromegaly, it remains unclear whether this is driven by GH excess or concurrent diabetes, as diabetic subgroups were not specifically analyzed.

#### 4.10.4. Diabetes-Related Complications

PAI-1 has been implicated in the progression of DN through modulation of TGF-β expression via the uPAR-mediated ERK/MAPK pathway, promoting renal extracellular matrix accumulation. A study showed that elevated PAI-1 levels have also been observed in patients with DR. Although PAI-1 is linked to coronary heart disease due to impaired fibrinolysis, its relationship with diabetic retinopathy is inconsistent, possibly reflecting organ-specific regulation and compensatory endothelial mechanisms in the retinal microvasculature [112]. However, serum and tear PAI-1 levels did not correlate significantly, which may be attributed to differing sample types or assay methods [113].

Given the involvement of PAI-1 in glucocorticoid-mediated metabolic dysfunction, additional studies are warranted to assess its therapeutic targeting potential, particularly in patients with overlapping endocrine disorders such as CS and acromegaly [114].

### 4.11. Thrombomodulin (TM) in GDM

Thrombomodulin (TM), a high-affinity thrombin receptor that acts as a natural anticoagulant, is present in the membrane of endothelial cells. As a cofactor, it helps thrombin activate protein C and prevents thrombin from functioning as a procoagulant. TM is also found in other cells (including keratinocytes, osteoblasts, macrophages, etc.) and may be involved in inflammation or cell differentiation. Because of enzymatic cleavage in the presence of cytokines, active neutrophils, and macrophages, soluble fragments of endothelium TM that circulate in the circulation are eliminated by urine. pTM level is regarded as a key molecular indicator of endothelial cell injury [12].

Studies assessing TM levels in GDM have reported conflicting findings. Some research has shown no significant differences in TM concentrations between GDM and non-GDM pregnancies [115]. Conversely, other studies suggest that TM levels may be more closely associated with long-term diabetes duration and renal dysfunction, rather than short-term glycemic fluctuations [116]. These discrepancies may stem from heterogeneous study populations, including participants with preexisting diabetes, thereby confounding GDM-specific interpretations. Future investigations should prioritize well-characterized cohorts and examine the longitudinal association between TM levels and microvascular complications in patients with GDM.

## 5. Clinical Implications and Practical Utility of Coagulation Biomarkers

Although this narrative review highlights numerous coagulation biomarkers associated with diabetes-related complications, their clinical applicability in terms of diagnosis, risk stratification, and disease monitoring remains limited owing to variability in study design and the absence of standardized reference values. Nonetheless, a few markers show consistent associations that may inform future clinical algorithms.

D-dimer, for example, is elevated in both T1DM and T2DM patients, particularly in patients with cardiovascular disease and nephropathy. Levels above 0.5 µg/mL fibrinogen equivalent units (FEUs) are typically considered abnormal, and some studies report >1.0 µg/mL as a cutoff indicating increased cardiovascular risk in diabetic patients [15]. However, diagnostic specificity remains low because of increases in numerous inflammatory and prothrombotic conditions.

Fibrinogen is another marker of interest. Concentrations exceeding 400 mg/dL have been consistently linked to poor glycemic control, increased platelet aggregation, and cardiovascular risk [31,32]. However, the lack of uniform threshold values limits its use in routine screening.

Despite frequent use in research, standardized cutoff values for coagulation biomarkers such as D-dimer and fibrinogen are lacking, limiting their clinical applicability in diabetes due to substantial inter-individual variability influenced by age, sex, ethnicity, and comorbid conditions; heterogeneity in assay methodologies and laboratory calibration across studies; dynamic fluctuations in biomarker levels due to acute-phase responses, infections, or transient hyperglycemia; and differences in study populations, including T1DM versus T2DM, or the presence of micro- versus macrovascular complications.

Von Willebrand factor reflects endothelial dysfunction and is elevated in diabetic retinopathy and nephropathy [13,75]. While elevated vWF may serve as an early marker of microvascular complications, interindividual variability and a lack of standard cutoff values hinder its diagnostic utility.

At present, limited data exist regarding the sensitivity, specificity, and cost-effectiveness of using these markers in routine clinical practice. Therefore, while promising, these biomarkers should currently be interpreted in conjunction with clinical findings and traditional risk factors. Their integration into standardized care protocols will require validation through large-scale prospective studies.

**Figure 5:**
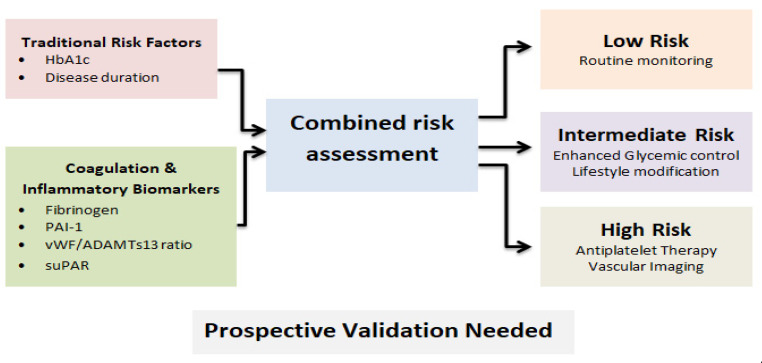
A hypothetical clinical algorithm integrating HbA1c, disease duration, and coagulation biomarkers such as fibrinogen, PAI-1, vWF/ADAMTS13, and suPAR could stratify patients by vascular risk and guide personalized preventive strategies. Prospective validation would be needed before clinical implementation.

Based on current evidence, when evaluated alongside traditional risk factors such as HbA1c, disease duration, blood pressure, and lipid profiles, selected coagulation and platelet biomarkers provide incremental predictive information for vascular complications in diabetes. In particular, fibrinogen and PAI-1 show the most consistent associations with macrovascular risk, reflecting systemic prothrombotic activity beyond conventional metabolic markers. The vWF/ADAMTS13 ratio captures endothelial dysfunction, while suPAR reflects residual inflammatory and immune activation not fully explained by standard clinical variables. In contrast, biomarkers such as D-dimer, tissue factor, and β-thromboglobulin remain largely exploratory, with inconsistent associations and limited evidence of additive predictive value. Conceptually, integrating higher-priority biomarkers with established risk factors may enable stratification of patients into vascular risk categories; however, this approach remains hypothetical and requires validation in prospective cohorts before clinical implementation (Figure 5).

Table 1 shows the differential expression of coagulation markers across various types of DM, highlighting the heterogeneity of hemostatic alterations. Table 2 summarizes key clinical insights into the selected coagulation markers discussed in this review.

## 6. Effect of Therapeutics on Coagulation Biomarkers in Diabetes Subtypes

Coagulation abnormalities are a hallmark of diabetes and contribute significantly to vascular complications.

In T2DM, interventions targeting coagulation biomarkers have shown varying degrees of therapeutic potential. Research has shown that elevated PAI-1 in T2DM promotes hypofibrinolysis and cardiovascular risk. Interventions such as biguanides (metformin), thiazolidinediones, and insulin sensitizers reduce PAI-1 levels and improve fibrinolytic balance, although direct PAI-1 inhibitors are not yet approved [118,119,120,121].

In another study reported that early intensive insulin therapy in T2DM improves endothelial dysfunction as measured by vWF, suggesting its potential utility for early risk stratification and monitoring therapy response [122]. İn addition, recombinant TM in experimental T2DM models protects pancreatic β-cells from apoptosis, improves glucose tolerance, and mitigates diabetic nephropathy through anti-inflammatory and antioxidant mechanisms [123,124,125]. TM also accelerates diabetic corneal and skin wound healing [126,127].

Furthermore, Interventions like insulin and metformin reduce P-selectin levels, improving platelet hyperreactivity in T2DM, and may reflect vascular health [128,129]. Limited evidence exists in T1DM; β-TG levels reflect platelet hyperactivity but do not consistently correlate with glycemic control [130]. suPAR is implicated in diabetic kidney injury and β-cell dysfunction, but clinical interventions (dapagliflozin) did not significantly modulate its levels [63,131]. Platelets from T2DM patients produce elevated TF, resistant to insulin inhibition, contributing to hypercoagulability [132]. Preclinical approaches, including TF pathway inhibitors, anti-TF antibodies, and microRNA-targeted therapies, show antithrombotic and anti-inflammatory effects, but clinical translation is limited by bleeding risk [133].

There is noticeable therapeutic gaps found across gestational, monogenic, and secondary diabetes that no studies were found evaluating coagulation biomarkers as direct therapeutic targets. While some TM studies in pregnancy and placental dysfunction suggest potential translational relevance [134,135], these are not diabetes-specific. In short, interventions targeting coagulation biomarkers show promising therapeutic potential in T2DM, but translation to other diabetes types remains largely unexplored. Evidence in T1DM and experimental models highlights mechanistic roles rather than clinically validated therapies. The effects of therapeutics on coagulation biomarkers across diabetes subtypes are summarized in Table 3.

**Table 3:** Effect of therapeutics on coagulation biomarkers in Diabetes subtypes.

Coagulation Marker	Type of Diabetes	Therapeutic Intervention	Effect/Outcome	Clinical and Preclinical Evidence
TF	T2DM	TF pathway inhibitors, anti-TF antibodies, microRNAs	Reduced platelet TF production; antithrombotic/anti-inflammatory effects in preclinical studies [132,133]	No clinical translation due to bleeding risk
PAI-1	T2DM	Metformin, TZDs, insulin sensitizers	Reduced PAI-1 levels; improved fibrinolysis [118,119,120,121]	Preclinical and clinical evidence; direct PAI-1 inhibitors not yet approved
tPA	T2DM	Metformin	Decreased PAI-1; improved fibrinolytic balance [118]	Limited human evidence
TM	T2DM	Recombinant TM, rTMD1, ETAR blocker (Atrasentan)	Reduced β-cell apoptosis, improved glucose tolerance, nephroprotection, enhanced wound/corneal healing [123,124,125,126,127]	Preclinical strong evidence; limited clinical data
vWF	T2DM	Intensive insulin therapy	Improved endothelial function; potential early marker [122]	Early-stage T2DM; monitoring utility suggested
P-selectin	T2DM	Insulin, Metformin	Reduced platelet hyperreactivity; improved vascular function [128,129]	Clinical evidence in T2DM only
β-TG	T1DM	Experimental: AN leaves extract, glyburide	Reduced platelet hyperactivity in rats; variable human correlation with glycemic control [130,136]	Limited therapeutic relevance; T1DM evidence mostly experimental
suPAR	T1DM, T2DM	Dapagliflozin	No significant change in serum suPAR levels [63,131]	Experimental and clinical trials; marker not suitable for therapy monitoring
TF, PAI-1, TM, vWF, P-selectin	GDM, Monogenic, Secondary Diabetes	None reported	N/A	Major knowledge gap

Abbreviations: T1DM, type 1 diabetes mellitus; T2DM, type 2 diabetes mellitus; GDM, gestational diabetes mellitus; PAI-1, plasminogen activator inhibitor-1; vWF, von Willebrand factor; suPAR, soluble urokinase plasminogen activator receptor; TZD, thiazolidinediones; TF, Tissue Factor; TM, Thrombomodulin; β-TG, β-Thromboglobulin.

## 7. Methodological Limitations and Evidence Strength

This narrative review synthesizes data from a wide range of studies with varying designs and methodological rigor. While efforts have been made to include recent and relevant publications, several limitations should be acknowledged. Most of the cited studies are observational, cross-sectional, or retrospective in nature, which inherently limits the ability to infer causality. Moreover, heterogeneity in study populations, sample sizes, outcome definitions, and analytical methods reduces the comparability and generalizability of findings. Confounding variables such as inflammation, medication use, glycemic variability, and comorbidities were not consistently adjusted for in many studies, possibly affecting the reported associations between coagulation markers and diabetes-related complications. Furthermore, standardized thresholds for biomarkers such as D-dimer, fibrinogen, or PAI-1 are lacking, making clinical application challenging.

Most available evidence is cross-sectional or observational, providing associations rather than causal or predictive insights. Future longitudinal and interventional studies are needed to determine whether coagulation biomarkers are active mediators or merely indicators of diabetic vascular risk. Given these limitations, the conclusions drawn from this review should be interpreted cautiously. While certain coagulation markers appear promising for risk stratification, their incorporation into routine clinical practice requires validation through well-designed prospective studies, randomized trials, and systematic reviews that assess the quality and strength of evidence.

## 8. Conclusions

DM is associated with significant alterations in coagulation and platelet function, contributing to an increased thromboembolic risk across multiple subtypes, including T1DM, T2DM, GDM, and monogenic and secondary forms of diabetes. In T1DM, endothelial dysfunction and platelet hyperreactivity are prominent, with elevated D-dimer, vWF, and P-selectin linked to microvascular and cardiovascular complications. T2DM is characterized by a more pronounced hypercoagulable state driven by insulin resistance and chronic low-grade inflammation, with increased fibrinogen, D-dimer, prothrombin, and PAI-1 levels contributing to both macrovascular and renal disease. GDM further amplifies the physiological hypercoagulability of pregnancy, while monogenic and secondary forms of diabetes exhibit heterogeneous coagulation profiles that reflect their underlying pathophysiology.

Collectively, coagulation and platelet activation biomarkers—including fibrinogen, D-dimer, PAI-1, vWF/ADAMTS13 ratio, and suPAR—provide valuable mechanistic and translational insights into the prothrombotic milieu of diabetes. However, despite their strong biological plausibility and consistent associations with vascular complications, their routine clinical screening cannot yet be recommended. This limitation is primarily due to the lack of standardized reference ranges, variable diagnostic specificity, and insufficient prospective validation across diverse diabetic populations.

Future longitudinal and interventional studies are required to establish clinically meaningful cutoff values, clarify the incremental predictive value of these biomarkers beyond established metabolic risk factors, and determine whether biomarker-guided strategies can meaningfully improve risk stratification and therapeutic decision-making. Until such evidence is available, coagulation biomarkers should be regarded as research and risk-enrichment tools, rather than components of routine clinical assessment.

This narrative review highlights both the shared and diabetes-specific perturbations of the coagulation cascade, underscoring the need for a cautious, evidence-driven approach to integrating hemostatic biomarkers into clinical practice.

## Figures and Tables

**Table 1:** Differential Expression of Coagulation Biomarkers in various Diabetes subtypes.

Diabetes Type	D-Dimer	Fibrinogen	PAI-1	vWF	P-Selectin	suPAR	β-TG	TM	Pathological Effects
T1DM	↑	↑/↔	↔	↑ (early DN)	↑	↑ (CVD risk)	↑	↔	Endothelial dysfunction
T2DM	↑↑	↑↑	↑↑	↑↑	↑	↑↑	↑	↑	Hypercoagulability + inflammation
GDM	↑	↑	↑	↑	↑	↑ (early predictor)	?	↔	Increased thrombosis risk during pregnancy
Monogenic DM	↔	↓	?	?	?	?	?	?	Minimal changes
Secondary DM	↑	↑	↑	↑	↑	?	?	↔	Hormone-induced coagulopathy

Footnote: Data were synthesized from peer-reviewed studies published between 2008 and 2026 evaluating hemostatic and endothelial biomarkers in different forms of diabetes. Arrows (↑/↓/↔) indicate the predominant direction of change relative to non-diabetic controls, based on the balance of available evidence. Variability among studies likely reflects methodological differences, patient selection, glycemic status, and treatment exposure. This table serves as a conceptual overview, not a quantitative meta-analysis. Abbreviations: T1DM, type 1 diabetes mellitus; T2DM, type 2 diabetes mellitus; GDM, gestational diabetes mellitus; DN, diabetic nephropathy; CVD, cardiovascular disease; D-dimer, fibrin degradation product; PAI-1, plasminogen activator inhibitor-1; vWF, von Willebrand factor; suPAR, soluble urokinase plasminogen activator receptor; β-TG, beta-thromboglobulin; TM, thrombomodulin; ↑, increased; ↑↑, markedly increased; ↓, decreased; ↔, unchanged; ?, insufficient or inconsistent data.

**Table 2:** Biomarker-Specific Pathophysiological Features, and Clinical Implications.

Biomarker	Pathophysiologic Role	Most Affected Diabetes Type	Clinical Implications	Key References
D-dimer	Fibrin degradation product reflecting thrombin generation and fibrinolysis	T2DM, DN progression	Predicts cardiovascular events and nephropathy severity	[30,117]
Fibrinogen	Acute-phase reactant promoting atherothrombosis	T2DM with CAD/ACS	Independent predictor of cardiovascular outcomes	[32,39,45]
PAI-1	Inhibits fibrinolysis; associated with insulin resistance and obesity	T2DM, obesity	High PAI-1 predicts incident diabetes and vascular risk	[106,107]
vWF	Marker of endothelial activation and damage	T2DM, CVD	Elevated levels indicate endothelial dysfunction	[76,77]
suPAR	Indicator of immune activation and endothelial injury	T1DM, T2DM (DKD)	Predicts CV events and eGFR decline	[62,63]

Abbreviations: T1DM, type 1 diabetes mellitus; T2DM, type 2 diabetes mellitus; DN, diabetic nephropathy; DKD, diabetic kidney disease; CAD, coronary artery disease; ACS, acute coronary syndrome; CVD, cardiovascular disease; eGFR, estimated glomerular filtration rate; PAI-1, plasminogen activator inhibitor-1; vWF, von Willebrand factor; suPAR, soluble urokinase plasminogen activator receptor.

## Data Availability

No new data generated or analyzed during this study are included in this article.
